# Versatile guardians: regenerative regulatory T cells in Parkinson’s disease rodent models

**DOI:** 10.1038/s41392-023-01681-4

**Published:** 2023-11-20

**Authors:** Chi Wang Ip, Jörg Wischhusen

**Affiliations:** 1 Department of Neurology, University Hospital of Würzburg, Würzburg, Germany; 2 Section for Experimental Tumor Immunology, Department of Obstetrics and Gynecology, University Hospital of Würzburg, Würzburg, Germany

**Keywords:** Neurological disorders, Diseases of the nervous system, Neuroimmunology

In a paper published in *Nature*, Park and colleagues have explored an innovative approach for improving adoptive neuron transfer, with possible implications for Parkinson´s Disease (PD) and other neurodegenerative diseases.^1^ By co-transplanting autologous regulatory T (Treg) cells, they managed to reduce surgical trauma-induced neuroinflammation, enhance the safety of cell replacement therapy by inhibiting proliferation of non-dopaminergic cells and improve therapeutic outcomes.

**Fig. 1:**
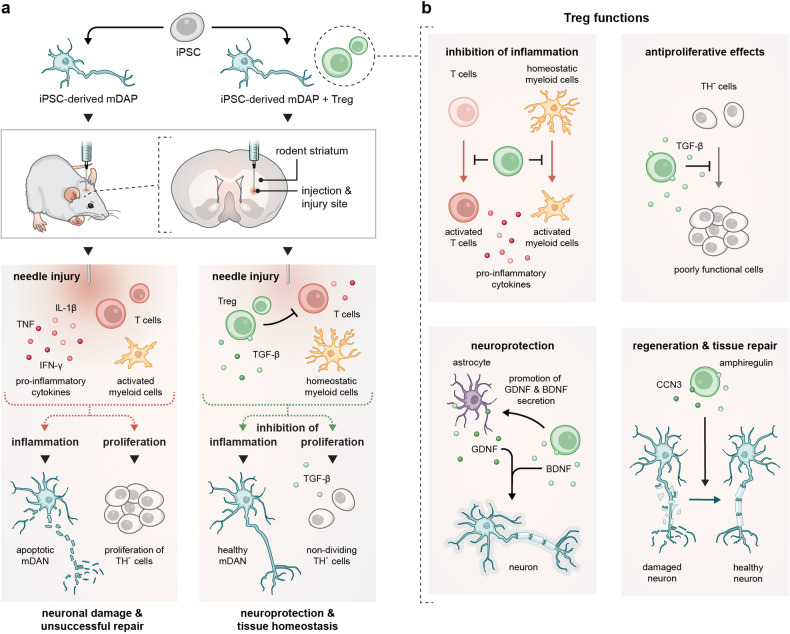
Regenerative regulatory T-cells rescue iPSC-derived dopaminergic neuron transplants in Parkinson’s disease rodent models*.* **a** Induced pluripotent stem cells (iPSCs) are transformed into midbrain dopaminergic progenitors (mDAP) and injected into the mouse striatum. The induced needle trauma, however, triggers inflammation. Local immune cells like microglia are thus activated and pro-inflammatory cytokines are released. This causes the loss of most transplanted tyrosine hydroxylase (TH)^+^ midbrain dopaminergic neurons (mDAN). In contrast, TH^-^ non-dopaminergic cells proliferate, resulting in scarring rather than neural repair. Injecting regulatory T cells (Treg) alongside mDAP prevents inflammation, allowing mDAN to survive. Moreover, proliferation of TH^-^ non-dopaminergic cells is prevented by Treg-derived TGF-β. **b** Treg are versatile in their protective function. Beyond having anti-inflammatory effects on local and recruited immune cells, they impair proliferation of mDAP-derived TH^-^ cells, i.e. cells that are no dopaminergic neurons and cannot repair the lesion. Treg further promote neuroprotection via BDNF and GDNF, and facilitate tissue regenerative processes via amphiregulin and CCN3. This figure was created by Sandy Westermann (scigraphix.com)

PD is a neurodegenerative disorder characterized by the loss of dopaminergic midbrain neurons in the *substantia nigra* (SN) and their projections into the striatum, leading to severe motor and non-motor symptoms. Recent advancements in stem cell technology raise the possibility that PD could eventually be treated by implanting dopaminergic neurons generated from human induced pluripotent stem cells (iPSc) and embryonic stem cells (ESC). However, significant limitations of this therapeutic approach are the low survival rate of transplanted neurons. Further, potential proliferation of unwanted cell populations gives rise to safety concerns (Fig. 1).

To overcome these limitations, Park et al. generated human midbrain dopaminergic progenitor (mDAP) cells, using human ESC, or skin-derived iPSC from a patient with PD as starting material. To induce lesions in the striatum of rodents, they stereotactically injected 6-hydroxydopamine (6-OHDA) into the SN. Subsequent intra-striatal injection of mDAP initially resulted in a very poor survival of implanted tyrosine hydroxylase-expressing (TH^+^) dopaminergic neurons. This was linked to needle-induced neuroinflammation. By co-transplanting autologous regulatory T cells (Treg), they then managed to reduce surgical trauma-related inflammation and improve therapeutic outcomes.

This study demonstrates that the surgical procedure of inserting a needle into the rodent striatum itself triggers profound neuroinflammation. Under inflammatory conditions, TH^+^ dopaminergic neurons demonstrated a poor survival rate (<10%) at two weeks after transplantation and in vitro, while almost all TH^-^ grafted cells survived. Mitigating neuroinflammation to prevent neuronal cell death thus appeared essential for successful cell transfer, even in autologous settings. Co-injecting CD4^+^CD25^+^FoxP3^+^ Treg, which are potent tolerogenic and anti-inflammatory cells required for immune homeostasis and tissue repair, was a logical, and successful next step. Still, while correct synapse formation by transplanted human iPSC-derived neurons and endogenous neurorepair processes require time, co-injected Treg have a limited lifespan. This may explain why the functionally impressive rescue in an amphetamine-induced rotation model in Fischer344 rats, observed at 20 weeks after implantation of human patient-derived mDAP cells, required additional daily treatment with cyclosporine A (CsA). Nevertheless, CsA treatment alone was significantly less effective than CsA treatment combined with adoptively transferred Treg.

This clearly indicates that Treg exert additional effects on top of CsA-induced immunosuppression. Moreover, beneficial effects of co-injected Treg were not only found in immunocompetent Fischer344 rats, or in mice that had been immunologically humanized with peripheral blood mononculear cells from the iPSC donor. (Of note, these mice ultimately die from graft-versus-host-disease, which may also be modulated by Treg). Treg were also protective in highly immunodeficient NSG mice and in vitro, when neurons were just exposed to proinflammatory cytokines such as IFN-γ, TNF-α, or IL1-β. Accordingly, effects of Treg must go beyond suppression of host inflammatory immune responses. Given that the safety of cell replacement therapy is of utmost importance, it is intriguing to note that Park et al. found that Treg inhibit the proliferation of undesirable cells. Indeed, the authors demonstrated that Treg-derived TGF-β limits the proliferation of TH^-^ cells, which also emerge from the injected iPSC- (or ESC-) derived mDAP. Still, some of the described findings imply further direct effects of Treg on the iPSC-derived dopaminergic neurons.

In this context, known regeneration-promoting mechanisms of Treg include (i) secretion of the growth regulatory protein CCN3, which in turn promotes differentiation of oligodendrocyte precursor cells and myelin regeneration,^2^ (ii) activation of the neuroprotective Rac1/Akt signaling pathway in dopaminergic neurons when CD47 on Treg binds to Signal regulatory protein α (SIRPα) on neurons, (iii) release of brain-derived neurotrophic factor (BDNF), of amphiregulin, TGF-β and IL-10, and (iv) polarization of astrocytes and microglia towards neuroprotective phenotypes characterized by secretion of BDNF and glial cell-derived neurotrophic factor (GDNF).^3^ Local administration of Treg, as performed in this study, allows for all these effects. In the MPTP mouse model for PD, adoptive i.v. transfer of Treg or in vivo Treg induction by treatment with recombinant granulocyte-macrophage colony-stimulating factor (GM-CSF) also protected nigral dopaminergic neurons - even without co-administration of mDAP. In a clinical phase I trial, treatment with rhGM-CSF (sargramostim) increased Treg frequency and function. Remarkably, this translated into improved scores on the Unified Parkinson’s Disease Rating (UPDRS-III) Scale and magnetoencephalography-recorded cortical motor activities in PD patients (NCT01882010).^3^

These remarkable effects of Treg in PD concur with recent clinical observations, which suggest that the persistent neuroinflammation observed in PD patients is no mere response to neurodegeneration and cell death. Instead, accumulation of pro-inflammatory pathological α-Synuclein, priming and expansion of α-Synuclein-specific T cells, infiltration of Th1-skewed CD4^+^ and activated CD8^+^ T cells into the SN and activation of microglia were found to precede the symptomatic loss of dopaminergic neurons.^4^ An increased frequency of effector memory T cells in peripheral blood correlates with more severe motor function impairments. A direct link with Treg was established when peripheral blood Treg from PD patients were found to possess reduced immunosuppressive capability and disease-associated functional deficits, which further contributes to pathological inflammation. Epidemiologically, a population-based case-control study including 48,295 PD patients and 52,324 controls found that intake of immunosuppressants like azathioprine and mycophenolate greatly reduced the risk of developing PD.^5^ As these medications shift the balance between effector and regulatory T cells, this study suggests that an early immunomodulatory intervention might interfere with the development of PD. Still, current symptomatic treatments for PD hardly modulate ongoing immune alterations.

In the present study, however, Park et al. used a model that resembled an advanced stage of disease, where Treg alone might not have been effective. (Apparently, Treg monotherapy was not tested.) Combining human mDAP, Treg and CsA, they observed a functional repair of the lesioned rat brain within 20 weeks, suggesting that adoptively transferred xenogeneic neurons survived and showed sufficient plasticity to integrate into pre-formed neural networks. In summary, this highlights the potential of a combined transplantation of stem cell-derived dopaminergic midbrain neurons and autologous anti-inflammatory Treg as a putative treatment strategy for PD patients. With this combined approach, Park et al. have successfully addressed crucial limitations of stem cell-based therapy. Importantly, as other post-mitotic neurons such as GABAergic neurons were similarly affected by needle trauma-induced neuroinflammation, similar strategies could also enhance the treatment of various other (neuro)degenerative diseases.
